# Modern risk stratification in coronary heart disease

**Published:** 2011-11-24

**Authors:** C. Ginghina, I. Bejan, C.D. Ceck

**Affiliations:** * "Carol Davila” University of Medicine and Pharmacy, Bucharest, Romania; ** Cardiology Department, “Prof. Dr. C.C. Iliescu” Institute for Cardiovascular Diseases, Bucharest, Romania

**Keywords:** cardiovascular risk, angina, myocardial infarction, acute coronary syndrome

## Abstract

The prevalence and impact of cardiovascular diseases in the world are growing. There are 2 million deaths due to cardiovascular disease each year in the European Union; the main cause of death being the coronary heart disease responsible for 16% of deaths in men and 15% in women. Prevalence of cardiovascular disease in Romania is estimated at 7 million people, of which 2.8 million have ischemic heart disease. In this epidemiological context, risk stratification is required for individualization of therapeutic strategies for each patient. The continuing evolution of the diagnosis and treatment techniques combines personalized medicine with the trend of therapeutic management leveling, based on guidelines and consensus, which are in constant update. The guidelines used in clinical practice have involved risk stratification and identification of patient groups in whom the risk-benefit ratio of using new diagnostic and therapeutic techniques has a positive value. Presence of several risk factors may indicate a more important total risk than the presence / significant increase from normal values of a single risk factor. Modern trends in risk stratification of patients with coronary heart disease are polarized between the use of simple data versus complex scores, traditional data versus new risk factors, generally valid scores versus personalized scores, depending on patient characteristics, type of coronary artery disease, with impact on the suggested therapy. All known information and techniques can be integrated in a complex system of risk assessment.

The current trend in risk assessment is to identify coronary artery disease in early forms, before clinical manifestation, and to guide therapy, particularly in patients with intermediate risk, which can be classified in another class of risk based on new obtained information.

**Abbreviations:** ACS = acute coronary syndrome; AMI = acute myocardial infarction; BNP = brain natriuretic peptide; BP = blood pressure; BPs = systolic blood pressure; CHD= coronary heart disease; CRP = C-reactive protein; CX= circumflex artery; EF= ejection fraction; LAD= left anterior descendending coronary artery; LV = feft ventricle; MI= myocardial infarction; NGAL= neutrophil gelatinase associated lipocalin; NT-proBNP = N-terminal pro B-type natriuretic peptide; RCA= right coronary artery; RV= right ventricle

## General Information

There are many classifications of known cardiovascular risk factors: major (showing a significant correlation with the risk of cardiovascular disease), associated (show a less significant correlation with cardiovascular risk); modifiable (can be controlled through diet / treatment) or not modifiable (i.e.: age, sex).[**1**] Classic risk factors are known: the INTERHEART study, which included the population in 52 countries, has identified nine risk factors responsible for over 90% of the myocardial infarction risk: smoking, dyslipidemia, hypertension, diabetes, obesity, diet, physical activity, alcohol consumption, psychosocial factors. [**2**]

A recent study [**3**] has proposed to demonstrate the prognostic impact of physical examination in patients with acute coronary syndrome. The result was that the integration of information obtained from physical examination in Killip classification is an important predictor of death from any cause in patients with acute coronary syndromes, of significant importance being age, Killip class, heart rate, systolic blood pressure and ST segment depression.

An example is the HNR (Heinz Nixdorf Recall) Study, which demonstrated the benefit of identifying subclinical atherosclerosis by using coronary calcium score, assessed by noninvasive CT, consisting of a high percentage of reclassification in the intermediate risk patient group and a more precise estimate of risk. Patients with intermediate risk (based on traditional risk factors according to Framingham score) were reclassified according to calcium score results: <100 in the low-risk class and > 400 in the high-risk class. [**4**]

## Risk Assessment Systems

A risk assessment system with a clinical utility must meet several criteria. It must use appropriate statistical methods, it must be performant (with internal and external validity), and it must include appropriate risk factors: with important discriminatory power, with prevalence in the target-population, and must be easy to use. [**5**]

Risk scores use proportional semiparametric (Cox) and parametric (Weibull) statistical risk models, complex methods such as cluster analysis and tree-structured analysis, or neural networks. [**5**] Risk scores’ performance is determined by the discrimination power (statistical parameters: AUROC and C Harrell test), calibration and reclassification (net reclassification index NRI).

Theoretical performance of a risk score is important, but in order to become a useful clinical tool must be accessible: simple, must contain available parameters and identify patients who might benefit from a specific treatment. [**6**] A comparative study on the prognostic value of risk scales in acute coronary syndromes demonstrated the superiority of GRACE and PURSUIT scores, which are complex, but are difficult to apply at the bedside, over the TIMI score, which is simpler, but more frequently used in clinical practice. [**7**]

The first step recommended in determining the patient’s risk is the calculation of the global cardiovascular risk, based on simple information, with the assessment of “classical” risk factors: age, sex, heredity, obesity, sedentary lifestyle, smoking, hypertension, diabetes mellitus, dyslipidemia, alcohol, psychosocial factors (i.e. Framingham, SCORE) and a careful clinical examination. The latter has an important prognostic value and constitutes the basis of several risk scores with wide application, such as the Killip classification in myocardial infarction. [**8**]

**Table 1:** Comparison of risk factors considered in the overall cardiovascular risk scores (according to [**9**])

	Framingham	SCORE	PROCAM (men)	Reynolds (men)
Risk factors	Age, sex, total cholesterol, HDL, smoking, BPs, antihypertensive medication	Age, sex, total cholesterol – HDL ratio, smoking, BPs	Age, LDL, HDL, smoking, diabetes, BPs, triglycerides, family history	Age, total cholesterol, HDL, smoking, BPs, CRP, family history of MI at age <60 years
Electronic address	http://hp2010.nhlbihi.net/atpiii/calculator	http://www.heartscore.org	http://www.chdtaskforce.com/coronary_risk_assessment.html	http://www.reynoldsriskscore.org/

Following initial assessment, patients are classified into three risk categories: low, intermediate or high, to guide therapeutic management. Thus, a patient classified as having a high risk will be subject to invasive investigations, without using additional methods to quantify risk. Instead, for a patient with intermediate risk, therapeutic decision can be influenced by the use of unconventional markers and modern, complex techniques, if they are able to reclassify the patient in a different class of risk: low or high. [**9**] New risk factors for atherosclerosis are described as: markers of inflammation, metabolic risk factors, thrombogenic factors, markers of subclinical atherosclerosis.

Risk assessment is a dynamic process.

### Coronary Heart Disease Risk Assessment

European Society of Cardiology suggests an algorithm for the evaluation of patients with acute coronary syndrome probability. The pain presence raises suspicion of acute coronary syndrome. The ECG may show ST segment elevation, normal pattern or atypical abnormalities. Depending on the biochemical markers (troponin) risk stratification can be made and therapeutic management can be guided. Afterwards, risk assessment will be made for secondary prevention.

#### Stable angina

Diagnosis of stable angina is primarily clinical and the leading role is the patient’s history. The most widely used classification is the Canadian Cardiovascular Society (CCS) Classification, describing four classes of risk depending on clinical features and circumstances of occurrence. [**10**]

**Table 2:** Canadian Cardiovascular Society Functional Classification of Stable Angina (according to [**10**])

Class	Canadian Cardiovascular Society Functional Classification
**I**	Ordinary physical activity does not cause angina, such as walking and climbing stairs. Angina with strenuous or rapid or prolonged exertion at work or recreation.
**II**	Slight limitation of ordinary activity. Walking or climbing stairs rapidly, walking uphill, walking or stair climbing after meals, or in cold, or in wind, or under emotional stress, or only during the few hours after awakening.Walking more than 2 blocks on the level and climbing more than one flight of ordinary stairs at a normal pace and in normal conditions.
**III**	Marked limitation of ordinary physical activity. Walking one to two blocks on the level and climbing one flight of stairs in normal conditions and at normal pace.
**IV**	Inability to carry on any physical activity without discomfort - anginal syndrome may be present at rest.

There are other classification systems: Duke Activity Specific Index or Seattle Angina Questionnaire, which can provide additional prognostic information [**11**], but in daily clinical practice they are less used.

The European Society of Cardiology [**12**] proposed a classification into three categories of annual cardiovascular mortality risk: low risk (<1%), intermediate risk (1-2%), elevated (>2%). Risk stratification requires integration of clinical parameters, stress test results, ventricular function and coronary angiography diagnosis. Individual prognosis of patients with stable angina is variable and the therapeutic management is different depending on the risk degree. Initial clinical evaluation (history, physical examination), rest electrocardiogram and laboratory tests can reveal unstable angina, in which case, the therapeutic management is specific. If the initial evaluation is suggestive of stable angina without signs of severity, the next step is the assessment of ischemia by using stress tests (stress ECG, echocardiography, scintigraphy), but, if there are characters of gravity: ECG changes, myocardial infarction, signs of heart failure, hypertension, diabetes mellitus, the cardiac performance is assessed by using the cardiac ultrasound or the MRI. Data integration allows patients classification in risk classes and modulation of therapeutic conduct: medical treatement with assessment of the therapeutic efficiency or coronary angiography, to determine the optimal revascularization formula.

The most important prognostic and survival factors in stable angina are left ventricular function, distribution and severity of coronary stenosis. [**13**]

Two forms of atypical angina are: Coronary X Syndrome and the vasospastic angina. Coronary X syndrome is defined by typical effort angina, positive stress tests and permeable epicardial coronary arteries [**14**] and was classified as a form of primary microvascular stable angina, with a good prognosis.[**15**] In vasospastic angina, the mortality risk is determined by the presence and severity of coronary artery stenosis [**16**] and the vascular hyperreactivity.

#### Acute coronary syndromes without ST segment elevation

Patients with acute coronary syndromes are a heterogenous group, with different risk of death or major cardiac events, and the initial assessment has an important role in the election of investigation and therapeutic decision.

The first classification in a group of risk in patients with unstable angina is made through history, depending on the character and frequency of anginal pain. Risk stratification in these patients can be performed by Braunwald classification of unstable angina in three risk classes.[**17**] (according to **Table 3**)

**Table 3:** Braunwald Classification of Unstable Angina (according to [**17**])

Class	Severity
**I**	New onset of severe angina or accelerated angina; no rest pain
**II**	Angina at rest within past month but not within preceding 48 h (angina at rest, subacute)
**III**	Angina at rest within 48 h (angina at rest, acute)
	**Clinical circumstances**
**A**	Develops in Presence of Extracardiac Condition That Intensifies Myocardial Ischemia (Secondary UA)
**B**	Develops in absence of extracardiac conditions (Primary UA)
**C**	Develops within 2 weeks of AMI (Postinfarction UA)

Clinical evaluation, ECG and biological makers allow the initial assignment of patients in one of the risk groups: high, intermediate or low. [**18**] (according to **Table 4**)

**Table 4:** Risk Stratification Based on Noninvasive Testing (according to [**18**])

Risk	Parameters
**High Risk **>3% annual mortality rate	1. Severe resting left ventricular dysfunction (LV EF < 35 %)
	2. High-risk treadmill score (score ≤ -11)
	3. Severe exercise left ventricular dysfunction (exercise LV EF < 35 %)
	4. Stress-induced large perfusion defect (particularly if anterior)
	5. Stress-induced multiple perfusion defects of moderate size
	6. Large, fixed perfusion defect with LV dilation or increased lung uptake (thallium-201)
	8. Echocardiographic wall motion abnormality (involving > two segments) developing at low dose of dobutamine (≤10 mg/kg/min) or at a low heart rate (<120 bpm)
	9. Stress echocardiographic evidence of extensive ischemia
**Intermediate Risk **1–3 % annual mortality rate	1. Mild/moderate resting left ventricular dysfunction (LV EF = 35–49%)
	2. Intermediate-risk treadmill score (-11 < score < 5)
	3. Stress-induced moderate perfusion defect without LV dilation or increased lung intake (thallium-201)
	4. Limited stress echocardiographic ischemia with a wall motion abnormality only at higher doses of dobutamine involving ≤ two segments
**Low Risk **< 1% annual mortality rate	1. Low-risk treadmill score (score ≥ 5)
****	2. Normal or small myocardial perfusion defect at rest or with stress
****	3. Normal stress echocardiographic wall motion or no change of limited resting wall motion abnormalities during stress

TIMI Score uses data on age, frequency of anginal episodes, risk factors and history of coronary artery stenosis, drug therapy (aspirin) and values of cardiac biomarkers. It is easy to calculate, each of the seven items of the questionnaire receiving 0 or 1 point, with a maximum score 7, and can be applied even in non-specialized areas.

**Table 5:** TIMI risk score (according to [**19**])

**Characteristics**	Age > 65 years
	At least 3 risk factors for CHD¹
	Significant coronary stenosis (eg, prior coronary stenosis ≥ 50%)
	ST deviation
	Severe anginal symptoms (eg, > 2 anginal events in last 24 h)
	Use of aspirin in last 7 days
	Elevated serum cardiac markers²
**Score**	**Rates of all-cause mortality, myocardial infarction, and severe recurrent ischemia prompting urgent revascularization through 14 days**
**0/1**	4,7%
**2**	8,3%
**3**	13,2%
**4**	19,9%
**5**	26,2%
**6/7**	40,9%
¹Risk factors include: family history of CHD, hypertension, hypercholesterolemia, diabetes, or being a current smoker;
²Creatine kinase MB fraction and/or cardiac-specific troponin level.

It has a superior prognostic value compared to solely using ECG and troponin [**20**], even if it has a less accurate predictive value of cardiovascular events.[**18**] TIMI score has been validated in unselected cases of suspected acute coronary syndrome, myocardial infarction with or without ST segment elevation, is used in analyzing tratement efficacy and identifying patients with different response to treatment.

GRACE risk score is based on the analysis of an international registry that included an unselected group of patients with acute coronary syndromes and evaluates clinical data, ECG and biological information: such as age, heart rate, systolic blood pressure, Killip class, the ECG changes, cardiac biomarkers, as well as two variables not previously identified: baseline creatinine level and cardiac arrest. This score has a significant discriminative power in assessing the risk of intra-hospital mortality, hospital discharge and at 6 months; patients are classified into three risk groups: low, intermediate and high. [**21,22**]

**Table 6:** In-hospital mortality and 6 months mortality according to GRACE risk score (according to [**22**])

Risk grade	Score	In-hospital mortality (%)
Low risk	≤ 108	< 1
Intermediate risk	109 - 140	1 – 3
High risk	> 140	> 3
**Risk grade**	**Score**	**Mortality at 6 months (%)**
Low risk	≤ 88	< 3
Intermediate risk	89 - 118	3 – 8
High risk	> 118	> 8

It is difficult for bedside use, but there are applications that facilitate automatic online calculating. Howerver, this is the optimum risk stratification score for daily practice, at admission and discharge. [**18,23**]

FRISC score is the only score that has a high capacity to identify patients who have a long-term benefit from an early invasive treatment strategy. [**18,24**]

PURSUIT score allows risk stratification in patients with unstable angina and myocardial infarction without ST segment elevation. [**25**]

Each investigation contributes to determining the patient’s degree of risk. Thus, biological analysis provides important information by evaluating traditional biomarkers, as well as novel markers. For risk stratification, the following markers can be used: of myocardial necrosis, inflammation, hemodynamic stress and neurohormonal activation, renal impairment, vascular injury and accelerated atherosclerosis (**Table 7**).

**Table 7:** Types of biomarkers used in risk stratification and choice of appropriate therapy in acute coronary syndromes (according to [**18**])

Markers	Example
Necrosis markers	Troponin
Inflammation markers	CRP, myeloperoxidase, pregnancy-associated plasma protein A, soluble ligand CD-40, interleukin 6
Hemodynamic stress and neurohormonal activation	BNP, NT-proBNP
Renal impairment	NT-proBNP, creatinine clearance, cystatin C, NGAL
Bioumoral particular context, vascular injury	Fibrinogen, platelet agregability
Accelerated atherosclerosis	Hemoglobin A1C

The most important marker for the diagnosis and early risk stratification is troponin, which allows the differentiation between unstable angina and myocardial infarction, without ST segment elevation; with an important prognostic value (high levels are correlated with a poor prognosis). [**26**] Dynamic evaluation of creatine-kinase and creatine-kinase MB has a high prognostic value, so brain natriuretic peptide (BNP) and C-reactive protein (CRP) are recommended for additional grading. Multiple studies have proven that BNP is an important prognostic biomarker in patients with acute coronary syndromes. C-reactive protein allows risk stratification, especially in patients grouped on the basis of traditional risk factors in the intermediate risk class [**27**], as an independent maker or in combination with troponin T value [**28**], but there are studies (RISCA), showing that it does not provide additional long-term prognostic information. [**29**]

It should be noted that the use of biological markers should be performed and interpreted in a clinical context. For example, homocysteine evaluation is not recommended in all patients to identify risk, but has additional value for patients with intermediate risk. [**28**]

We are now trying to identify the prognostic value and the limits of individual markers and association of different investigations to allow data integration into risk scores (similar to clinical scores), “multi-marker strategy” to achieve a better risk grading [**18**], mentioning that there are also limits to be known (the synergistic effect of markers).

The modern concept of risk stratification is a “dynamic risk model”, [**29**] representing the parallel evolution of risk markers with the disease, their dynamic interpretation. This is why the reassessment of risk after the first day and at discharge is of great importance.

#### Acute coronary syndromes with ST segment elevation

##### Risk stratification in acute coronary syndromes with ST segment elevation - pre-hospital

Myocardial infarction with ST segment elevation has a high mortality rate, especially in the first 2 hours after onset, before patients reach hospital, and the reasons are: the incidence of malignant arrhythmias, ignoring or misinterpreting different types of haemodynamic instability, lack of early coronary reperfusion procedures [**30**], so that the quick, correct diagnosis and risk assessment of these patients pre-hospital is essential

Risk assessment in pre-hospital occurs mainly based on clinical, electrocardiographic and paraclinical data. The following clinical characteristics suggest an increased vital risk: age over 70 years, signs of tissue hypoperfusion, systolic blood pressure <120 mmHg, tachycardia > 110 bpm or bradycardia <60 bpm, psychomotor agitation, pulmonary rales or protodiastolic gallop. [**30**]

**Table 8:** Clinical criteria for assessing risk in patients with acute myocardial infarction - pre-hospital (according to [**30**])

Parameter	Low risk	High risk
Age (years)	< 70	> 70
AV (bpm)	>60 , < 110	<60, >110
TAs (mmHg)	> 120	< 120
Pulmonary rales	No	Yes
3rd Sound	No	Yes
Signs of shock	No	Yes

The parameters mentioned above allow the risk grading in Killip class, a simple classification and widely used in clinical practice. It also allows the identification of hemodynamic profile: normodinamic, hiperdinamic syndrome, hypotension, bradycardia, hypovolemia, cardiac pump failure, RV myocardial infarction, cardiogenic shock. [**30**]

**Table 9:** Hemodynamic profiles in patients with acute myocardial infarction (according to [**30**])

Hemodynamic profile	Dyspnoea	SBP mmHg	AV bpm	Teguments	Cianosis	Jugular veins	Rales	Diuresis
Normodynamic	-	N	N	N	-	N	-	N
Hyperdynamic	-	>140	>120	N	-	N	-	N
Hypotension-bradycardia	-	<95	<60	N	-	N	-	N
Hypovolaemia	-	<95	>100	N	-	N	-	N/oliguria
Pump failure	+/++/+++	N/<95	N/>100	N/cold	-	N/turg	+/++/+++	N/oliguria
RV infarction	-/+	N/<95	>100	cold	++	N/turg	-/+	N/oliguria
Cardiogenic shock	+++	<95	>100	cold	+++	N/turg	+++	N/oliguria
N= normal

The ECG provides data for risk stratification in acute coronary syndromes with ST segment elevation electrocardiogram, and can often be done pre-hospital, allowing Topol grading.

Paraclinical risk criteria are the arterial oxygen saturation reduction < 90% [**2**], a parameter easily obtained by the use of pulse oximetry, hyperglycemia > 120 mg / dl (correlated to 3 fold increased risk of death)[**31**], increased levels of troponin in the first 6 hours after symptoms onset. [**32**]

An important aspect is the early initiation of therapy in patients with myocardial infarction with ST segment elevation in pre-hospital with antiplatelets, anticoagulants and even fibrinolytic medication. In this case risk assessment is essential to identify patients with bleeding and increased bleeding risk, represented by: bleeding disorders, history of ulcer disease or gastrointestinal bleeding, anemia without a known cause, severe hypertension, history of ischemic or hemorrhagic stroke (including transient), signs of senile dementia, recent head trauma, recent surgery (last three weeks), body weight below 65 kg. [**33,34**]

##### Risk stratification in acute coronary syndromes with ST segment elevation - in hospital

Initial assessment at hospital admission of patients with acute coronary syndromes with ST segment elevation should use two risk scales: clinical (Killip) and ECG (Topol). Killip Classification (1967) is still useful in clinical practice even if the impact on mortality has decreased due to enlargement of the therapeutic arsenal. [**35**]

**Table 10:** Killip classification of acute myocardial infarction (modified after [**34,35**])

Class	Clinic	“Historical” mortality (%)	Mortality at 30 days (GUSTO-1) (%)
**I**	No rales, no 3rd heart sound	8,4	5,1
**II**	Pulmonary congestion with rales < 50% of the lung fields or 3rd heart sound	30,5	13,6
**III**	Pulmonary oedema with rales > 50% of the lung fields	44	32,2
**IV**	Cardiogenic shock	82,1	57,8

The ECG allows rapid risk evaluation of patients with myocardial infarction with ST segment elevation, including them into one of the five Topol classes, the most reserved prognosis being for class Topol I. [**36**]

**Table 11:** Topol Classification of MI based on ECG at admission correlated with angiographic data (according to [**36**])

	Occlusion location	ECG at admission	Mortality at 30 days	Mortality at 1 year
1. LAD -proximal	Before the first septal perforating artery	↑ST in DI, aVL, V1-V6 and fascicular block or bundle branch block	19,6%	25,6%
2. LAD - medium	After the first septal erforating artery, before the great diagonal artery	↑ST in DI, aVL, V1-V6	9,2%	12,4%
3. LAD - distal or Diagonal artery	After the great diagonal artery or first diagonal leasion	↑ST in V1-V4 or ↑ST in DI, aVL, V5-V6	6,8%	10,2%
4. Inferior moderate MI (RV, posterior, lateral)	Proximal RCA or CX	↑ST in DII, DIII, aVF and any or all of the following: a) V1, V3R, V4R b) V5-V6 c) R>S in V1-V2	6,4%	8,4%
5. Inferior small MI	Distal RCA or CX or CX branches	↑ST in DII, DIII, aVF	4,5%	6,7%

Location of ECG changes, ST segment elevation amplitude and QRS duration are electrocardiographic features with prognostic value, correlated with the risk of adverse events.[**37**] ST segment elevation in aVR derivation has a controversial prognostic value, but a value >1.5 mm associated with anterior myocardial infarction and a value >1 mm associated with inferior myocardial infarction, in patients without intraventricular conduction disturbances, is associated with increased mortality at 30 days.[**38**] Markers with predictive value of myocardial revascularization success are described: terminal QRS distorsion (grade 3 ischemia) on the initial ECG is an independent predictor marker for coronary reperfusion absence after primary angioplasty.[**39**]

Mortality risk in patients with AMI can be assessed according to clinical and hemodynamic parameter values: cardiac index and pulmonary capillary pressure (obtained by right cardiac catheterization), according to Forrester classification, rarely used today. Normal hemodynamic profile includes patients with normal cardiac output (> 2.2 l/m/m2) and pulmonary capillary wedge pressure < 18 mmHg, increased pressure in the pulmonary capillary under a normal cardiac output corresponds to patients with acute pulmonary edema, patients with hypovolemic status presents low cardiac output and pulmonary capillary pressure at low, while patients in cardiogenic shock present a low cardiac output in the presence of increased pressure in the pulmonary capillary.[**40**]

Summing up the information provided by clinical and ECG data, the most important independent prognostic markers are older age, higher Killip class, and presence of tachycardia, hypotension and anterior location of the myocardial infarction.[**41**] Independent predictive value also have: history of a myocardial infarction, time to treatment, height, weight, diabetes and smoking.[**42**] Initial echocardiographic evaluation brings information on the location and extent of the myocardial infarction; LV systolic function was correlated with long-term prognosis in these patients.[**43**]

Risk assessment in acute coronary sindrom with ST segment elevation is a continuous process, so that each diagnostic step brings additional information and allows a dynamic stratification of the patients’ risk. Coronary angiography allows assessing the coronary anatomy, coronary lesions, the best therapeutic option, the opportunity and myocardial revascularization modality.

**Table 12:** Indications for myocardial revascularization modality of choice (surgical versus interventional) (according to [**44**])

Coronary anatomy	CABG	PCI
1VD or 2VD – non-proximal LAD	IIbC	IC
1VD or 2VD – proximal LAD	IA	IIa B
3VD simple lesions, full functional revascularization achievable with PCI, SYNTAX score ≤22	IA	IIa B
3VD complex lesions, incomplete revascularization achievable with PCI, SYNTAX score > 22	IA	III A
Left main (isolated or 1VD, ostium/shaft)	IA	IIa B
Left main (isolated or 1VD, distal bifurcation)	IA	IIb B
Left main + 2VD or 3VD, SYNTAX score ≤ 32	IA	IIb B
Left main + 2VD or 3VD, SYNTAX score ≥33	IA	III B

Currently, there are several risk stratification scores based on angiographic results (**Table13and14**), but information on the comparative value of such scales of risk is limited, each using populations with different characteristics, the results being reported at different, not-superimposable distances of time.[**44**]

In case of interventional revascularization, it is important to grade the coronary flow (TIMI) and the myocardial blush (MBG- a semiquantitatively parameter dependent on the tissular phase of the myocardial perfusion), which are important prognostic factors.

**Table 13:** Grading of coronary flow (according to [**45**])

Grade	Definition
**0 : no perfusion**	- no antegrade flow beyond the point of occlusion
**1 : penetration with minimal perfusion**	- the contrast material passes beyond the area of obstruction, but "hangs up" and fails to opacify the entire coronary bed distal to the obstruction for duration of the cine run
**2 : partial perfusion**	-the contrast material passes across the obstruction and opacifies the coronary bed distal to the obstruction; the rate of entry of contrast material into the vessel distal to the obstruction or its rate of clearance from the distal bed (or both) are perceptibly slower than its entry into or clearance from comparable areas not perfused by the previously occluded vessel, e.g., the opposite coronary artery or the coronary bed proximal to the obstruction.
**3 : complete perfusion**	- antegrade flow into the bed distal to the obstruction occurs as promptly as antegrade flow into the bed proximal to the obstruction, and clearance of contrast material from the involved bed is as rapid as clearance from an uninvolved bed in the same vessel or the opposite artery

**Table 14:** Grading of myocardial blush (according to [**46**])

**MBG 0**	no myocardial blush or contrast density; myocardial blush persisted ("staining"): leakage of the contrast medium into the extravascular space
**MBG 1**	minimal myocardial blush or contrast density
**MBG 2**	moderate myocardial blush or contrast density but less than that obtained during angiography of a contralateral or ipsilateral non–infarct-related coronary artery
**MBG 3**	normal myocardial blush or contrast density, comparable with that obtained during angiography of a contralateral or ipsilateral non–infarct-related coronary artery

After the primary angioplasty in acute myocardial infarction, the no-reflow phenomenon can be seen in 10 to 40% of cases [**41**], characterized by the presence of coronary TIMI flow <3 or TIMI 3 but 0-1 myocardial blush, negative prognostic marker, correlated with a prolonged duration of myocardial ischemia, severe arrhythmias, hemodynamic deterioration, increased risk of mechanical complications.

After reperfusion therapy, it is necessary to identify patients at high risk for reinfarction or death. In this respect it is important to assess infarct size and LV function at rest in the first 24-48 hours (if not performed previously to angioplasty). It is necessary to assess myocardial viability: using myocardial perfusion scintigraphy (with thallium-201 or technetium-99m), stress echocardiography (usually dobutamine), MRI or PET, as well as evaluating the risk of arrhythmia and to initiate sudden death prevention. Patients without symptomatic arrhythmias and with EF > 60% are at low risk, so you do not need further investigation. High risk patients have EF < 40%, heart failure symptoms, non-sustained ventricular tachycardia, sustained monomorphic ventricular tachycardia induced at electrophysiological study. Other risk factors are: T wave alternation, heart rate variability, QT dispersion, baro-reflex sensitivity, signal-gated ECG. [**41**]

Risk evaluation in patients with acute coronary syndromes is dynamic, with reassessment after each diagnostic or therapeutic step, with impact on the investigation plan, therapeutic management, with identification of immediate and long-term prognosis.

Modern risk stratification in coronary artery disease involves a complex approach (incorporating data and taking into account traditional risk factors unconventional) and personalized.
